# Copper Tolerance Mediated by *FgAceA* and *FgCrpA* in *Fusarium graminearum*

**DOI:** 10.3389/fmicb.2020.01392

**Published:** 2020-06-26

**Authors:** Xin Liu, Yichen Jiang, Dan He, Xin Fang, Jianhong Xu, Yin-Won Lee, Nancy P. Keller, Jianrong Shi

**Affiliations:** ^1^Jiangsu Key Laboratory for Food Quality and Safety-State Key Laboratory Cultivation Base, Ministry of Science and Technology/Key Laboratory for Control Technology and Standard for Agro-product Safety and Quality, Ministry of Agriculture and Rural Affairs/Key Laboratory for Agro-product Safety Risk Evaluation (Nanjing), Ministry of Agriculture and Rural Affairs/Collaborative Innovation Center for Modern Grain Circulation and Safety/Institute of Food Safety and Nutrition, Jiangsu Academy of Agricultural Sciences, Nanjing, China; ^2^Department of Medical Microbiology and Immunology, University of Wisconsin-Madison, Madison, WI, United States; ^3^School of Food and Biological Engineering, Jiangsu University, Zhenjiang, China; ^4^College of Food Science, Tibet Agriculture and Animal Husbandry University, Nyingchi, China; ^5^School of Agricultural Biotechnology, Seoul National University, Seoul, South Korea; ^6^Department of Bacteriology, University of Wisconsin-Madison, Madison, WI, United States

**Keywords:** *Fusarium graminearum*, copper tolerance, *FgAceA*, copper transporters, metallothionein, virulence

## Abstract

All organisms must secure essential trace elements (e.g., Cu) for survival and reproduction. However, excess trace element accumulation in cells is highly toxic. The maintenance of copper (Cu) homeostasis has been extensively studied in mammals, bacteria, and yeast but not in plant pathogens. In this study, we investigated the molecular mechanisms of copper tolerance in *Fusarium graminearum*, the important wheat head scab fungus. RNA-seq revealed induced expression of the P-type ATPase transporter *FgCrpA* and metallothionein (MT) *FgCrdA* after excess Cu treatment. Deletion of *FgCrpA* but not *FgCrdA* resulted in reduced tolerance to Cu toxicity. The “Cu fist” transcription factor FgAceA was involved in Cu detoxification through activation of *FgCrpA*. △FgAceA was more sensitive to copper toxicity than △FgCrpA and overexpression of *FgCrpA* restored copper tolerance in △FgAceA. *FgAceA* negatively regulated aurofusarin production and its biosynthetic gene expression. △FgCrpA and △FgAceA were reduced in virulence in flowering wheat heads and synthesized decreased amounts of the mycotoxin deoxynivalenol when challenged with excess Cu. Taken together, these results suggest that mediation of Cu tolerance in *F. graminearum* mainly relies on the Cu efflux pump and that *FgAceA* governs Cu detoxification through activation of *FgCrpA*.

## Introduction

Copper (Cu) is an essential trace element that cycles between Cu^1+^ (reduced) and Cu^2+^ (oxidized) states in virtually all organisms (Dalecki et al., 2017; Gerwien et al., 2018). By coordinating with proteins and serving as a redox cofactor, Cu confers changes in protein structure, catalytic activity and protein-protein interaction, thus controlling a variety of cellular biochemical processes (Kim et al., 2008). However, these same properties can be highly detrimental to microorganisms when Cu is present in excess. Free Cu can lead to oxidative stress by reacting with reactive oxygen species (ROS) to generate hydroxyl radicals that damage many biomolecules, including DNA, membrane lipids and proteins (Kim et al., 2008). Furthermore, Cu can disrupt the active sites of metallothioneins (MTs), such as iron-sulfur enzymes, and displace other metals from their cognate enzymes, leading to their inactivation (Besold et al., 2016).

Given its toxic properties to microorganisms, Cu-containing compounds have been historically used as antimicrobials both in healthcare and agriculture (Dollwet and Sorenson, 1985; Grass et al., 2011; Festa et al., 2014; Michels et al., 2015). There is an emerging concept in innate immunity that animal hosts intentionally exploit copper toxicity as a weapon to combat invading microbes. For example, macrophages can attack invading microbes with high Cu levels, and Cu is elevated at sites in the lung and serum during infection (Besold et al., 2016).

In the host-microbe battle, on the other hand, the microbial intruder is equipped with a battery of Cu detoxification defenses that promotes survival and colonization in the host, including Cu binding MTs and Cu exporting P-type ATPases to defend against toxic levels of Cu. MTs are cysteine (Cys)-rich low molecular weight polypeptides found in organisms from prokaryotes to mammals (Ding et al., 2014). MTs are utilized in response to high Cu levels and detoxify Cu through binding the Cys of Cu via Cys-thiolate bonds (Palacios et al., 2011; Babula et al., 2012). P-type ATPases are heavy metal translocators conserved in all biological kingdoms that regulate intercellular Cu levels (Odermatt et al., 1993; Solioz and Odermatt, 1995; Ladomersky and Petris, 2015; Antsotegi-Uskola et al., 2017; Wiemann et al., 2017).

In the model yeast *Saccharomyces cerevisiae*, the metallo-regulation of the Cu-binding transcription factor (TF) Ace1 functions in Cu-replete cells and regulates the expression of Cu detoxification-related genes, including those encoding MTs (*Cup1* and *Crs5*) and Cu/Zn super oxide dismutase, SOD (*SOD1*) under excess Cu conditions (Keller et al., 2005; Cyert and Philpott, 2013). In the human fungal pathogen *Cryptococcus neoformans*, *CMT1* and *CMT2* genes encode cysteine-rich Cu-binding MTs, whose expression levels are dramatically upregulated in response to elevated Cu levels, play essential roles in Cu detoxification (Ding et al., 2011) and are critical for fungal virulence (Ding et al., 2013). While MTs have been proven to be the major players in Cu detoxification in *S. cerevisiae* and *C. neoformans*, P-type ATPases that pump Cu extracellularly through the transmembrane channel are utilized for Cu export in *Candida albicans* and *Aspergillus* spp. In the pathogenic yeast *C. albicans*, *CRP1*, which encodes a plasma membrane-localized Cu exporter P-type ATPase (Crp1), is transcriptionally upregulated by high Cu conditions in an Ace1-dependent manner, and deletion of *CRP1* resulted in a Cu-sensitive growth phenotype as well as massive intracellular Cu accumulation (Weissman et al., 2000). Recent studies have shown that *A. fumigatus, A. nidulans*, and *A. flavus* share a similar Cu detoxification machinery; under excess Cu conditions, the Cu-binding TF *AceA* induces the expression of the P-type ATPase *CrpA* for Cu detoxification (Antsotegi-Uskola et al., 2017; Wiemann et al., 2017; Cai et al., 2018; Yang et al., 2018). Both *AceA* and *CrpA* are virulence determinants in the human pathogen *A. fumigatus* (Wiemann et al., 2017). Recently, Yang et al. illustrated that in the crossover pathogen *A. flavus*, deletion of P-type ATPases (*A. flavus* contains two, CrpA and CrpB) and the TF *AceA* resulted in significantly reduced virulence in mice but had no impact on virulence in maize seeds (Yang et al., 2018).

*Fusarium graminearum* species complex is the major causal agent of Fusarium head blight (FHB) or scab of wheat and barley, which is among the most important diseases of cereal crops worldwide (Bai and Shaner, 2004; Goswami and Kistler, 2004). Epidemics of FHB not only cause huge yield losses but also contaminate grains with harmful trichothecene mycotoxins, threatening the health of both humans and animals (Lombaert et al., 2003; Rasmussen et al., 2003; Tutelyan, 2004; Isebaert et al., 2005; Ji et al., 2014). Despite in-depth studies of copper homeostasis in human pathogens, information on the Cu detoxification machinery and the roles of this machinery in the virulence of plant pathogens is still limited, and thus we examined the possible existence and importance of Cu-exporting mechanisms in the wheat pathogen *F. graminearum.*

The objectives of this study were to identify and characterize the major factors determining copper detoxification and their possible regulatory mechanisms in *F. graminearum*. Our results indicate that copper detoxification in the wheat pathogen *F. graminearum* relies mainly on Cu export similar to that in *C. albicans* and *Aspergillus* spp.

## Materials and Methods

### Strains, Culture Media, and Growth Conditions

*Fusarium graminearum* strain PH-1 (NRRL 31084) (Cuomo et al., 2007) was used as the parental wild-type. To assess the mycelial growth, the wild-type strain PH-1 and its corresponding mutants were grown on solidified FMM plates (Fusarium minimal medium) (Leslie and Summerell, 2007) amended under different Cu and/or menadione concentrations and incubated at 25°C. Fungal biomass was compared by collecting mycelia from 3-day-old liquid FMM culture incubated at 25°C in a shaker (180 rpm). For conidiation, five 5 mm mycelial plugs of the wild type strain PH-1 and its mutants taken from the edge of a 3-day-old colony were inoculated in a 150 ml triangular flask containing 50 ml of CMC (carboxymethyl cellulose) medium and incubated at 25°C, 180 rpm for 3 days in a shaker with light (Iida et al., 2008; Chen et al., 2018). The number of conidia in CMC medium was counted using a hemacytometer. Conidial germination rates were compared by re-suspending conidia in 2% sucrose solutions amended with or without 20 μM Cu at 25°C for 4 or 6 h. Each experiment was carried out in triplicate and repeated for three times.

### Nucleic Acid Manipulation, PCR Primers, Mutant Generation, and Confirmation

Using double-joint PCR strategy (Yu et al., 2004), deletion cassettes for each targeted gene were generated and transformed into the wild type PH-1 by using polyethylene glycol (PEG)-mediated protoplast transformation method (Proctor et al., 1995b). PCR primers used in this study were synthesized by Sangon Biotech (Shanghai, China) and are listed in Supplementary Table S1. Genes were deleted by creating constructs where 5′ and 3′ flanking regions were fused to the hygromycin gene *HPH* obtained from pBlueScript-hph or the G418 sulfate gene *NEO* from pBlueScript-neo. Solidified FMM supplemented with hygromycin (100 mg/L) or G418 sulfate (100 mg/L) was used to select transformants. PCR screened-positive transformants were single spore isolated and stored in 15% glycerol at −80°C for further experiments. To construct FgAceA^c^ or FgCrpA^c^, the full-length DNA of *FgAceA* or *FgCrpA*, including their promoter region (about 1.0 kb), was cloned into the pYF11 plasmid (Bruno et al., 2004), and then the recombinant plasmid pYF11-FgAceA or pYF11-FgCrpA was transformed into its corresponding deletion mutant. Southern blot hybridization analysis of the mutants and its wild-type strain was performed using a detection starter kit II according to the manufacturer’s instructions (Roche Diagnostics, Mannheim, Germany).

### Construction of Phylogenetic Tree

Predicted protein sequences released in FungiDB^1^ were aligned with Clustal W (Thompson et al., 1994) and a phylogenetic tree was constructed with MEGA 5.10 software using a neighbor-joining method (Kumar et al., 2008).

### RNA Extraction and Gene Expression Analysis

Conidia (10^5^) of *F. graminearum* wild-type strain PH-1 and its corresponding mutants were inoculated into 50 ml liquid FMM in triplicates and cultured at 25°C, 180 rpm for 3 days in a shaker. Mycelia was collected by filtering through sterile miracloth, washed with sterile water and then one half mycelia was grown under the same conditions with the other half shifted to a Cu treatment for several time points. Mycelia was then collected, washed with sterile water, frozen with liquid nitrogen and lyophilized for 24 h. Total RNA of *F. graminearum* strains was extracted using TaKaRa RNAiso Plus (TaKaRa, Dalian, China) and cDNA was reverse transcripted using TaKaRa PrimeScript^TM^ RT Master Mix (TaKaRa, Dalian, China). For quantitative Real-Time PCR (qRT-PCR) assays, expression of genes was determined with primer pairs listed in Supplementary Table S1. qRT-PCR amplifications were performed in a LightCycler^®^ 96 (Roche Molecular Systems, Inc.) using the TaKaRa SYBR^®^ Premix Ex Taq^TM^ II (Tli RNaseH Plus) (TaKaRa, Dalian, China).

For RNA-seq analysis, 10^5^ conidia of the wild-type strain PH-1 was inoculated into 50 ml liquid FMM without Cu and cultured at 25°C with shaking (180 rpm) for 3 days with a subsequent shift to Cu-treated condition (100 μM) for 1 h with triplicates as described above. RNA samples were prepared to perform digital transcriptome analysis by the RNA-seq approach (Shanghai Majorbio Bio-pharm Technology Co., Ltd.). The data were analyzed on the free online platform of Majorbio I-Sanger Cloud Platform^2^.

### Copper Quantification

Conidia (10^5^) of *F. graminearum* wild-type strain PH-1 and corresponding mutants were inoculated into 50 ml liquid FMM in four replicates and cultured at 25°C, 180 rpm for 3 days in a shaker. Subsequently, Cu was added to one half of the culture at a final concentration of 100 μM and fungi further cultured for 12 h. Mycelia were then collected, washed by sterile water, and lyophilized. Quantification of Cu from *F. graminearum* strains was carried out according to the method previously described by Shanghai Microspectrum Chemical Technology Service Co., Ltd. (Yang et al., 2018).

### Virulence Tests

Conidia of *F. graminearum* strains were harvested from 4-day-old CMC cultures, re-suspended in 0.01% (vol/vol) Tween 20 and adjusted to 1 × 10^5^ conidia/ml. Virulence tests were performed using a single floret injection method as previously described (Wu et al., 2005). Briefly, 10 μl of conidial suspension (1 × 10^5^/ml) was injected into a single floret in the central spikelet of single flowering wheat heads of susceptible cultivar Zhenmai 10 at early anthesis with or without 3 g/L Cu fungicide (Nordox Cuprousoxide) spraying. Ten spikes were used for each strain. Infected spikelets in each inoculated wheat head were recorded and photographed 15 days after inoculation. The experiments were repeated three times.

### DON Production Assays

Conidia (10^5^) of *F. graminearum* wild-type strain PH-1 and corresponding mutants were inoculated into 30 ml trichothecene biosynthesis inducing (TBI) liquid with or without Cu in three replicates and cultured at 28°C for 7 days. The filtrate and fungal mass were then collected separately, frozen with liquid nitrogen and lyophilized for 24 h. The filtrate was re-dissolved in methanol and fungal mass was measured. The amount of DON in each sample was determined using a high-performance liquid chromatography-mass spectrometer/mass spectrometer (HPLC-MS/MS) system (Shimadzu 30A LC system coupled to a Triple Quad 6500 plus, Sciex, United States). Mass spectrometric parameters were according to a previously described method (Dong et al., 2016). The experiment was repeated two times.

### Statistical Analysis

All data were presented as the means ± standard deviation (SD). Statistical significance analysis was performed between the wild type parent PH-1 and the deletion mutants with group *t*-tests with software package SPSS (version 13 for Windows, 2004). A *p*-value less than 0.05 was labeled as statistically significant.

## Results

### Genes Involved in Cu Tolerance Revealed by RNA-Seq in *F. graminearum*

To gain a genomic perspective of the Cu tolerance mechanism in *F. graminearum*, we treated the wild-type strain PH-1 with or without 100 μM CuSO_4_ for 1 h. Subsequently, RNA was extracted and prepared for RNA sequencing (RNA-Seq). All RNA-Seq raw data were deposited at the National Center for Biotechnology Information (NCBI) Sequence Read Archive (SRA)^3^ under bioproject accession number PRJNA601796.

Overall, by comparing RNA-Seq data, we found that genes encoding putative Cu transporters were significantly differentially regulated following 100 μM CuSO_4_ treatment. In *F. graminearum*, six genes encoding putative Cu-importing transporters, including the *S. cerevisiae* high-affinity Cu transporter Ctr1p ortholog FGRAMPH1_01G09843, the *S. cerevisiae* high-affinity Cu transporter Ctr2p homologs FGRAMPH1_01G19393 and FGRAMPH1_01G23855, the *A. fumigatus* Cu transporter CtrA1 orthologs FGRAMPH1_01G10595 and FGRAMPH1_01G13479, and the *S. cerevisiae* intracellular Cu ATPase Ccc2p ortholog FGRAMPH1_01G09315, were significantly downregulated (Supplementary Table S2). Furthermore, the *C. albicans* Cu-exporting ATPase *Crp1* ortholog *FgCrpA* (FGRAMPH1_01G10037) and the *C. albicans* MT *Crd2* ortholog *FgCrdA* (FGRAMPH1_01G09281) were significantly upregulated (Supplementary Table S2). Based on the observations from the RNA-Seq data, we next characterized the possible roles of the *F. graminearum* Crp1 ortholog (FgCrpA) and the Crd2 ortholog (FgCrdA) in Cu detoxification.

Previous studies have shown that the “Cu fist” DNA binding domain protein AceA was crucial in mediating Cu tolerance in *Aspergillus* spp. by activating the Cu-exporting transporters. The *S. cerevisiae* Ace1/YGL166W ortholog FgAceA (FGRAMPH1_01G09843) was identified using BlastP. Phylogenetic analysis showed that the Cu-exporting ATPase FgCrpA, the MT FgCrdA, and the “Cu fist” DNA binding domain protein FgAceA shared high level of sequence identities with their orthologs in yeasts and *Aspergillus* spp. (Supplementary Figures S1A–C).

### Expression Patterns of *FgCrpA* and *FgCrdA* in Response to Cu Toxicity

**FIGURE 1:**
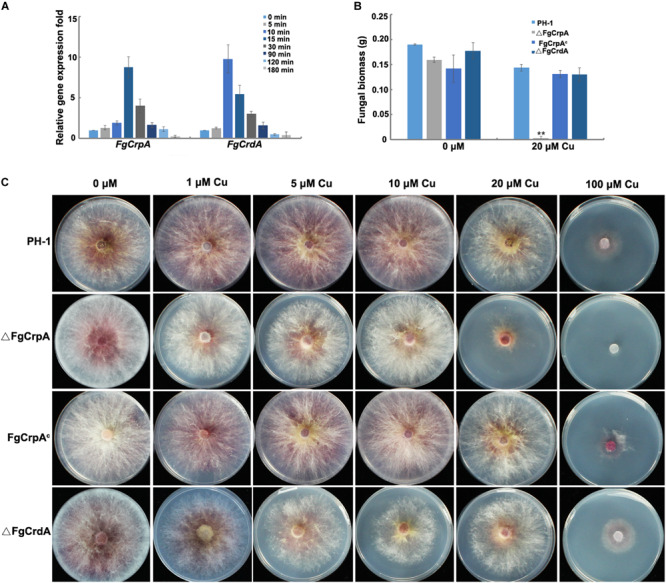
*FgCrpA* and *FgCrdA* were induced by Cu treatment and their roles in Cu detoxification. **(A)** Expression of *FgCrpA* and *FgCrdA* in *F. graminearum* after Cu treatment analyzed by quantitative Real-time PCR assays. The wild-type strain PH-1 was grown in liquid FMM (Cu free) for 3 days at 25°C and then collected, washed using sterile water and transferred to liquid FMM supplemented with CuSO_4_ at 100 μM for different time courses. **(B)** Fungal growth was measured by determining the dry weight of the wild-type strain and mutants grown in 50 ml liquid FMM amended with or without CuSO_4_ at 20 μM in a shaker at 180 rpm at 25°C for 3 days. Bars indicated means and error bars denote standard deviation from three repeated experiments. A *t*-test was performed to determine significant differences, **p < 0.01. **(C)** Phenotypic characterization of △FgCrpA, FgCrpA^c^, and △FgCrdA under Cu stress, equal numbers of conidia (2 × 10^3^) were spot inoculated onto solidified FMM amended with CuSO_4_ at different concentrations indicated in the figure and cultured at 25°C for 3days.

Quantitative real-time PCR (qRT-PCR) results showed that the expression of *FgCrpA* and *FgCrdA* in *F. graminearum* was induced rapidly by exposure to 100 μM Cu. *FgCrpA* reached its maximal expression level after exposure to Cu treatment at 15 min, followed by a decrease in expression (Figure 1A). *FgCrdA* was also induced by Cu treatment, and its expression displayed a similar pattern as *FgCrpA*; the maximal expression level was observed at 10 min, followed by a decrease (Figure 1A).

### *FgCrpA* but Not *FgCrdA* Plays an Essential Role in Cu Tolerance in *F. graminearum*

To determine the roles of *FgCrpA* and *FgCrdA* in Cu tolerance in *F. graminearum*, we generated the deletion mutants, △FgCrpA and △FgCrdA, which were further confirmed by diagnostic PCR and Southern blot (Supplementary Figures S2, S3). Deletion of *FgCrpA* resulted in reduced tolerance to Cu concentrations of 20 μM or higher (Figures 1B,C). However, no obvious difference in Cu tolerance was noticed between △FgCrdA and the wild-type PH-1 (Figures 1B,C). Further, there were no additive Cu sensitivity effect observed in the △FgCrdA△FgCrpA double mutant as compared with △FgCrpA (data not shown). Transformation of the full-length *FgCrpA* with its promoter region into △FgCrpA rescued tolerance to Cu (Figures 1B,C).

### The Cu-Binding TF *FgAceA* Regulates *FgCrpA* During Cu Detoxification in *F. graminearum*

**FIGURE 2:**
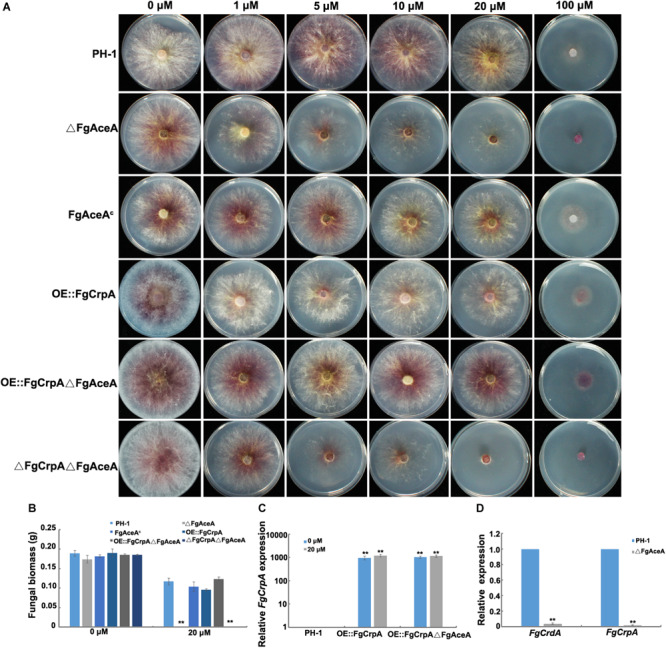
Phenotypic analysis of △FgAceA and its regulation on *FgCrpA* and *FgCrdA*. **(A)** Phenotypic characterization of the wild type strain PH-1 and mutants under Cu stress, equal numbers of conidia (2 × 10^3^) were spot inoculated onto solidified FMM amended with CuSO_4_ at different concentrations indicated in the figure and cultured at 25°C for 3 days. **(B)** Fungal growth was measured by determining the dry weight of the wild type strain PH-1, △FgAceA, FgAceA^c^, OE:FgCrpA, OE:FgCrpA△FgAceA, and △FgCrpA△FgAceA grown in liquid FMM amended with or without CuSO_4_ at 20 μM in a shaker at 180 rpm at 25°C for 3 days. A *t*-test was performed to determine significant differences, **p < 0.01. **(C)** Relative expression of *FgCrpA* in OE:FgCrpA or OE:FgCrpA△FgAceA analyzed by qRT-PCR assays. **(D)** Relative expression of *FgCrpA* and *FgCrdA* in △FgAceA analyzed by qRT-PCR assays.

The “Cu fist” DNA binding domain protein AceA has been identified in several *Aspergillus* spp. and proven to regulate the response to excess Cu by activating the Cu-exporting transporters CrpA (and CrpB in *A. flavus*). In contrast, in *Aspergillus* the MT, CrdA, is not activated by AceA (Wiemann et al., 2017; Yang et al., 2018). To explore the roles of the *F. graminearum AceA* ortholog *FgAceA* in mediating Cu detoxification, an *FgAceA* full-length deletion strain, ΔFgAceA, was generated and confirmed by diagnostic PCR and Southern blot (Supplementary Figure S4). Deletion of *FgAceA* resulted in even more Cu sensitivity compared to ΔFgCrpA. ΔFgAceA barely grew on FMM supplemented with 5 μM Cu. The double mutant ΔFgCrpAΔFgAceA was more sensitive than ΔFgAceA when the FMM was treated with 20 μM Cu (Figures 2A,B). Complementation with the full-length *FgAceA* driven by its native promoter restored Cu tolerance to that of wild type (Figures 2A,B).

To investigate the regulatory mechanism of *FgAceA* in *F. graminearum* Cu tolerance, we generated an overexpression strain of *FgCrpA* (OE:FgCrpA) by replacing its native promoter with the constitutive promoter *gpdA* from *A. nidulans* in both the wild-type strain PH-1 and ΔFgAceA. OE:FgCrpA strains were screened by diagnostic PCR and further confirmed by Southern blot (Supplementary Figure S5) and qRT-PCR. The transcriptional level of *FgCrpA* was significantly upregulated with or without Cu treatment (Figure 2C). OE:FgCrpA exhibited a restored Cu tolerance in the ΔFgAceA background (Figures 2A,B), however, OE::FgCrpA in the wild-type strain PH-1 did not exhibit an increased Cu tolerance (Figure 2A). Deletion of *FgAceA* ameliorated the induced *FgCrpA* and *FgCrdA* expression after excess Cu exposure (Figure 2D).

### Deletion of *FgAceA* and *FgCrpA* Caused Defects in Conidial Formation and Germination Under Excess Cu Conditions in *F. graminearum*

**FIGURE 3:**
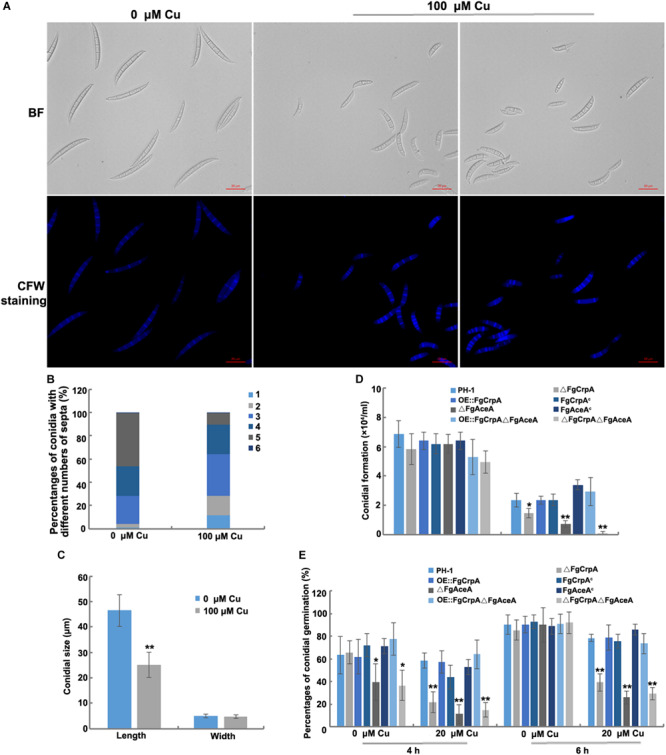
Excess Cu treatment caused defects in conidial morphogenesis. Differences in *F. graminearum* conidial morphology **(A)**, numbers of septa **(B)**, and conidial size **(C)** between conidia treated and untreated with Cu at 100 μM. **(D)** Conidial formation was measured by inoculating five mycelial plugs taken from wild-type strain PH-1 and its mutants in 50 ml liquid CMC amended with or without CuSO_4_ at 20 μM in a shaker with light at 180 rpm at 25°C for 3 days. **(E)** Conidial germination was also compared between wild-type strain PH-1 and its mutants by re-suspending the conidia in 10 ml liquid FMM amended with or without CuSO_4_ at 20 μM in a shaker with light at 180 rpm at 25°C for 4 h and 6 h. A *t*-test was performed to determine significant differences, *p < 0.05, **p < 0.01.

*F. graminearum* produced atypical conidia with shorter sizes and fewer septa when cultured under excess Cu conditions (Figures 3A–C). Conidial formation and germination were significantly reduced in ΔFgCrpA (*p* < 0.05), ΔFgAceA (*p* < 0.01) and ΔFgCrpAΔFgAceA (*p* < 0.01) under 100 μM Cu treatment (Figures 3D,E). The double deletion mutant ΔFgCrpAΔFgAceA was more sensitive to Cu as reflected by greater impairment of both conidial formation and germination compared with both single deletion mutants (Figures 3D,E). Noticeably, deletion of *FgAceA* caused reduced conidial germination even without excess Cu challenge at 4 h but was restored to wild-type level when the germination time was extended to 6 h (Figure 3E).

### Altered *FgAceA* Expression Affects Aurofusarin Accumulation in *F. graminearum*

**FIGURE 4:**
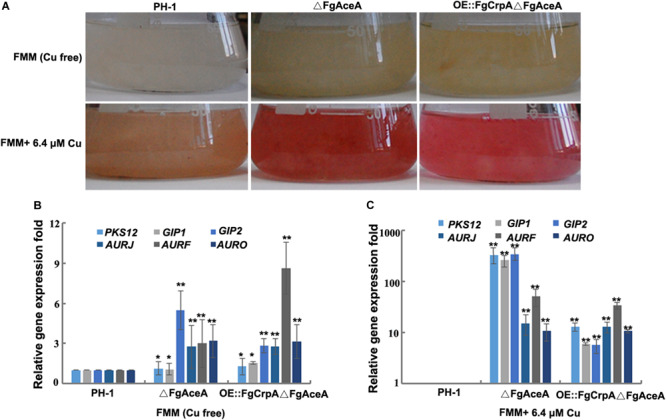
Cu homeostasis affects aurofusarin accumulation in *F. graminearum*. **(A)** *F. graminearum* wild-type strain PH-1, △FgAceA, or OE:FgCrpA△FgAceA was cultured in 50 ml liquid FMM with or without 5 μM (trace element level) Cu in a shaker with light at 180 rpm at 25°C for 4 days. Relative expression of six genes involved in aurofusarin biosynthesis (*PKS12*, *GIP1*, *GIP2*, *AURJ*, *AURF*, and *AURO*) in △FgAceA or OE:FgCrpA△FgAceA when grown in 50 ml liquid FMM with **(B)** or without 5 μM (trace element level) **(C)** Cu in a shaker with light at 180 rpm at 25°C for 4 days. A *t*-test was performed to determine significant differences, *p < 0.05, **p < 0.01.

One immediately observed phenotype of ΔFgAceA grown in high Cu medium was an intense red pigmentation (Figure 4A). We hypothesized that this pigment was the known secondary metabolite aurofusarin and confirmed this likelihood by the dense red color produced. qRT-PCR results which showed that in liquid FMM with or without Cu, the expression of six genes (*PKS12*, *GIP1*, *GIP2*, *AURJ*, *AURF*, and *AURO*) involved in aurofusarin biosynthesis increased dramatically in Δ*FgAceA*, and trace amounts of Cu stimulated the expression of these six genes (Figure 4B).

### Reduced Cu Tolerance Caused by *FgAceA* or *FgCrpA* Deletion Aggravates ROS Sensitivity in *F. graminearum*

**FIGURE 5:**
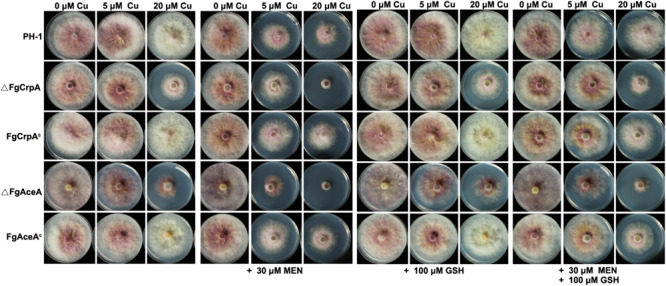
Reduced Cu tolerance caused by *FgAceA* or *FgCrpA* deletion aggravates ROS sensitivity in *F. graminearum*. Growth of *F. graminearum* strains on Cu-containing FMM media added with supplements (menadione, MEN and/or _L_-glutathione, GSH) at various concentrations as indicated at 25°C for 4 days.

Previous studies have shown that excess Cu could generate reactive oxygen species such as hydroxyl radicals (Ding et al., 2013, 2014). To test whether Cu detoxification was involved in mediating reactive oxygen intermediates (ROI) stress in *F. graminearum*, wild type strain PH-1, ΔFgCrpA, ΔFgAceA and their complementary strains were challenged with excess Cu and menadione. Results showed that 30 μM menadione and increasing Cu had inhibitory effects on all the *F. graminearum* strains tested. Reduced Cu tolerance caused by deletion of *FgAceA* or *FgCrpA* aggravates ROS sensitivity as demonstrated by the reduced growth of ΔFgCrpA and ΔFgAceA when grown on Cu containing FMM media supplemented with 30 μM menadione (Figure 5). Moreover, the reductant L-glutathione (GSH) could relieved the severe phenotype of all strains (Figure 5), suggesting that compromises in the Cu detoxification pathway aggravates ROI toxicity.

### Reduced Cu Tolerance Hinders Virulence and DON Biosynthesis in *F. graminearum*

**FIGURE 6:**
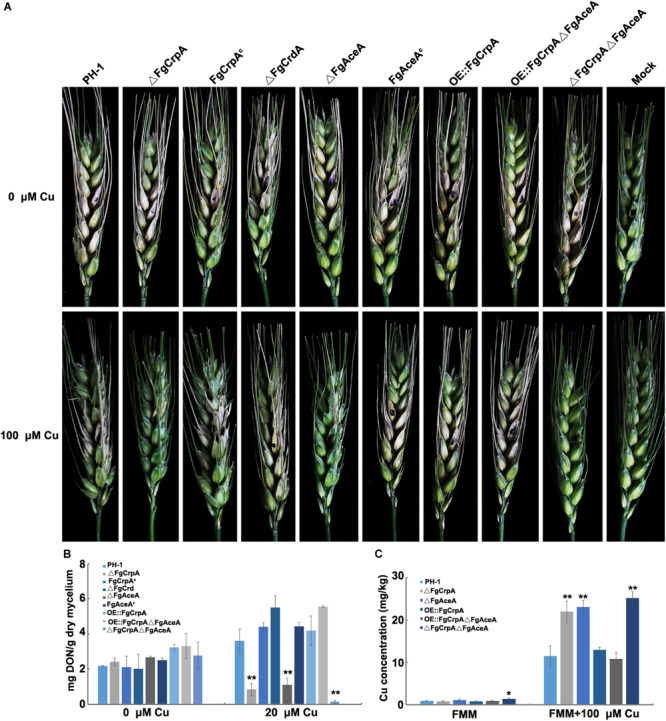
Comparisons of virulence, DON accumulation and Cu concentration between wild-type strain PH-1 and its mutants. **(A)** Difference in virulence of *F. graminearum* strains inoculated on Cu treated and untreated flowering wheat heads. **(B)** Difference in the ability of DON accumulation virulence of *F. graminearum* strains inoculated in 30 ml liquid TBI (inoculum: 10^5^ spores) with or without 20 μM Cu and cultured at 28°C for 7 days. **(C)** Difference in Cu concentration in mycelium collected from the wild-type strain PH-1 and its mutants treated or untreated with 100 μM Cu for 12 h. A *t*-test was performed to determine significant differences, *p < 0.05, **p < 0.01.

Pathogenicity assay results showed that the ability to invade untreated flowering wheat heads was not impaired by the single or double deletion of *FgAceA* and *FgCrpA*, as shown in Figure 6A (upper panel). However, when inoculated onto Cu fungicide-treated flowering wheat heads, ΔFgCrpA, ΔFgAceA, and ΔFgCrpAΔFgAceA could not successfully colonize the inoculated spikelet, thus completely losing their aggressiveness (Figure 6A, lower panel). Reintroduction of *FgCrpA* or *FgAceA* to their respective deletion mutants and overexpression of *FgCrpA* in ΔFgAceA rescued their lost virulence on Cu fungicide-treated flowering wheat heads (Figure 6A, lower panel). Deletion of *FgCrdA* did not result in visible changes in virulence in *F. graminearum* regardless of Cu treatment (Figure 6A).

The toxic secondary metabolite deoxynivalenol (DON) produced by *F. graminearum* is a known virulence factor during plant infection (Proctor et al., 1995a; Ilgen et al., 2008). The reduced Cu tolerance caused by disruption of *FgAceA* and/or *FgCrpA* also affected DON accumulation. As shown in Figure 6B, ΔFgCrpA, ΔFgAceA, and ΔFgCrpAΔFgAceA produced significantly (*p* < 0.01) lower amounts of DON under 20 μM Cu treatment than did the wild-type strain PH-1. Reintroduction of *FgCrpA* or *FgAceA* to their respective deletion mutants and overexpression of *FgCrpA* in ΔFgAceA rescued DON biosynthesis. Deletion of *FgCrdA* did not result in changes in DON biosynthesis in *F. graminearum*. When treated with 20 μM Cu, the *F. graminearum* wild-type strain PH-1 accumulated more DON than the untreated TBI liquid (Figure 6B).

Next, we determined the Cu concentration in the mycelium and compared the differences between the mutants and their wild-type strain PH-1. The results showed that when cultured in FMM liquid (trace level of Cu), only ΔFgCrpAΔFgAceA showed significantly (*p* < 0.05) increased cellular accumulation of Cu compared to the wild-type strain PH-1 (Figure 6C). However, when cultured in excess Cu (FMM liquid amended with 100 μM), the Cu concentration in ΔFgCrpA, ΔFgAceA, and ΔFgCrpAΔFgAceA increased significantly (*p* < 0.01) compared with the wild-type strain PH-1 or the complementary strain. Additionally, the overexpression of *FgCrpA* in ΔFgAceA rescued the Cu export deficiency of the mutant, and the Cu level decreased to that of the wild-type (Figure 6C).

## Discussion

Accumulating evidence suggests that during infection, invading fungal pathogens will be confronted with elevated levels of essential trace nutrients harnessed by the host, collectively known as “nutritional immunity” (Ballou and Wilson, 2016). Moreover, fungal pathogens have also evolved an accurate mechanism to balance the fine lines between the essentiality and toxicity of these essential trace nutrients to survive and colonize within the infected host. For example, all living organisms maintain Cu homeostasis by regulating the balance between Cu uptake, utilization and detoxification (Ding et al., 2014; Ballou and Wilson, 2016). In this study, we characterized the copper tolerance determinants in the important wheat head blight fungus *F. graminearum*. Overall, our study showed that *F. graminearum* Cu resistance is mainly mediated by the ATP-based export system and not by a Cu MT.

The P-type ATPase *FgCrpA* and the MT *FgCrdA* were both found to be significantly upregulated when treated with excess Cu in RNA-Seq analysis and further verified by qRT-PCR. This was consistent with observation in *Aspergillus* spp. Cu-induced *CrpA* and *CrpB* expression was confirmed in *A. flavus* by using Northern Blot analysis (Yang et al., 2018). In *A. nidulans*, Cu-induced CrpA and CrdA protein expression was confirmed by Western blot (Antsotegi-Uskola et al., 2017). As Crp and Crd shared conserved expression pattern and functions in response to Cu toxicity, we expected protein expression of FgCrpA and FgCrdA to be induced in *F. graminearum*. The results of gene disruption assays indicated crucial roles of *FgCrpA* but not *FgCrdA* in mediating Cu resistance in *F. graminearum*, indicating that Cu efflux is the predominant mechanism of Cu detoxification rather than MT-mediated Cu buffering. Together with previous studies carried out in other fungi, including *C. albicans* and *Aspergillus* spp. (*A. fumigatus*, *A. flavus*, *A. nidulans*), these results indicated that ATP-based efflux, as the principal Cu detoxification mechanism, is not unique and likely a well-conserved feature among filamentous fungal species (Antsotegi-Uskola et al., 2017; Wiemann et al., 2017; Cai et al., 2018; Yang et al., 2018).

Apart from Cu efflux, MT-related buffering also plays critical roles in Cu detoxification for some fungi. MTs function as Cu storage proteins by chelating excess Cu in yeast species. In *S. cerevisiae*, cells are protected from excess Cu by activating the MT-encoding genes *cup1* and *crs5*, and surplus Cu is then coordinated (Ecker et al., 1986; Culotta et al., 1994; Palacios et al., 2011; Thiele, 2015). In the human fungal pathogen *Cryptococcus neoformans*, the Cu metallo-regulatory TF Cuf1 activates the Cu-buffering MT-encoding genes *Mt1* and *Mt2* for Cu detoxification (Ding et al., 2013; Garcia-Santamarina et al., 2017). In the soil organism *F. oxysporum*, the Cu metallothionein Mt1 has been shown to be involved in mediating resistance to metal toxicity and virulence (Lorenzo-Gutiérrez et al., 2019). Despite the important roles of MTs in Cu detoxification in yeast species and *F. oxysporum*, their homologs in other filamentous fungi, including *A. flavus*, *A. fumigatus*, and *A. nidulans*, have been reported to be not particularly important. Deletion of *CrdA* caused no significant differences in Cu tolerance in these *Aspergillus* spp. In *F. graminearum*, although *FgCrdA* has been identified to respond to excess Cu treatment, its disruption did not alter Cu tolerance in △FgCrdA, the exact role of *FgCrdA* in Cu detoxification remains to be explored. Alternatively, Cu binding secondary metabolites – possibly filling a MT-like function – are hypothesized to be involved in Cu homeostasis in filamentous fungi (Raffa et al., 2019).

Cu homeostasis is tightly regulated in *F. graminearum* by the Cu-responsive TF *FgAceA*. We showed that deletion of *FgAceA* resulted in reduced Cu tolerance and ceased the induction of *FgCrpA* expression by excess Cu loading, suggesting that *FgCrpA* is transcriptionally expressed in an FgAceA-dependent manner under excess Cu stress. Overexpression of *FgCrpA* in △FgAceA could compensate for the reduced Cu tolerance, indicating that *FgAceA* mediates Cu homeostasis by regulating the expression of the copper exporting P-type ATPase *FgCrpA*. In *S. cerevisiae*, Ace1 functions by recognizing and binding to the Cu-dependent responsive element ACE in the promoter regions of its target MT-encoding genes *cup1* and *crs5* and the superoxide dismutase gene *sod1*. ACE contains the core sequence 5′-HTHXXGCTGD-3′ (D = A, G, or T; H = A, C or T; and X = any residue). The promoter region of *FgCrpA* contains one core ACE sequence (TTATGCTGT), indicating a potential FgAceA binding domain.

Cu could not be properly transported out of the cell and was accumulated in mutants lacking *FgAceA* or *FgCrpA* (Figure 6C); thus, these mutants displayed compromised phenotypes when challenged with excess Cu, as expected. Conidial formation and germination as well as DON biosynthesis and the ability to colonize flowering wheat heads were impaired in △FgAceA and △FgCrpA when treated with high levels of Cu. We found that *FgAceA* negatively regulates aurofusarin biosynthesis. In both liquid FMM medium with and without trace amounts of Cu, the accumulation of the red pigment aurofusarin increased significantly in △FgAceA compared with its wild-type parent. Six genes involved in aurofusarin biosynthesis were significantly upregulated in △FgAceA and OE:FgCrpA△FgAceA (Figures 4B,C). Overexpression of *FgCrpA* in △FgAceA did not restore the overproduction of aurofusarin, suggesting that *FgAceA* has additional roles in negatively regulating secondary metabolism and bypassing the mediation of Cu tolerance.

*FgAceA* and/or *FgCrpA* deletion mutants could not colonize on Cu fungicide-treated flowering wheat heads. Several reasons could account for the virulence loss of the mutants. First, it could not be separated from the defects in conidial germination and reduced aerial hyphae under excess Cu treatment. Second, ROS such as hydroxyl radicals generated with excess Cu or plant oxidative burst could also be responsible for the unsuccessful colonization of the mutants. Additionally, DON has been identified as a crucial virulence factor and plays an important role in aggressiveness of the fungus among the spikelets of the infected wheat head, the decreased DON production in the mutants challenged with excess Cu may also account for the loss of full virulence.

In summary, we elucidated the copper tolerance mechanism in the important wheat head blight fungus *F. graminearum*, which is mainly mediated by the ATP-based extrusion system. The P-type ATPase *FgCrpA* is transcriptionally regulated by the Cu-fist TF *FgAceA*, and both are crucial for Cu tolerance.

## Data Availability Statement

The datasets presented in this study can be found in online repositories. The names of the repository/repositories and accession number(s) can be found in the article/ Supplementary Material.

## Author Contributions

XL and YJ carried out the experimentation of this work. JS and NK conceived the experiments and wrote the manuscript. DH, XF, and JX helped create strains for this research. Y-WL helped revise this manuscript. All authors contributed to the article and approved the submitted version.

## Conflict of Interest

The authors declare that the research was conducted in the absence of any commercial or financial relationships that could be construed as a potential conflict of interest.

## Footnotes

1 http://fungidb.org/fungidb/

2 www.i-sanger.com

3 https://www.ncbi.nlm.nih.gov/sra

## Supplementary Material

FIGURE S1 Bioinformatic analysis of putative Cu detoxification determinants.

FIGURE S2 Diagram for generation and verification for △FgAceA by Southern blotting analysis.

FIGURE S3 Diagram for generation and verification for ΔFgCrpA by Southern blotting analysis.

FIGURE S4 Diagram for generation and verification for △FgCrdA by Southern blotting analysis.

FIGURE S5 Diagram for generation and verification for OE:FgCrpA by Southern blotting analysis.

TABLE S1 PCR primers used in this study.

TABLE S2 Expression of genes encoding putative copper-binding proteins revealed by RNA-seq.
