# Supplementary material for: Copper Tolerance Mediated by FgAceA and FgCrpA in Fusarium graminearum
Source: Front Microbiol. 2020 Jun 26;11:1392. doi: 10.3389/fmicb.2020.01392 (PMC7333239; doi:10.3389/fmicb.2020.01392)
Supplement: FIGURE S1 — Bioinformatic analysis of putative Cu detoxification determinants. [file Data_Sheet_1.docx]

**Supplementary Information**

**FIGURE S1 |** Bioinformatic analysis of putative Cu detoxification determinants. The transcription factors AceA (**A**), ATPase transporters CrpA (**B**) and metallothioneins CrdA (**C**) in fungi. The phylogenetic trees were generated using the neighbor-joining method as described in Material and Methods. Bootstrap values were shown in the figure and were analyzed with 1000 replicates.

**A** **
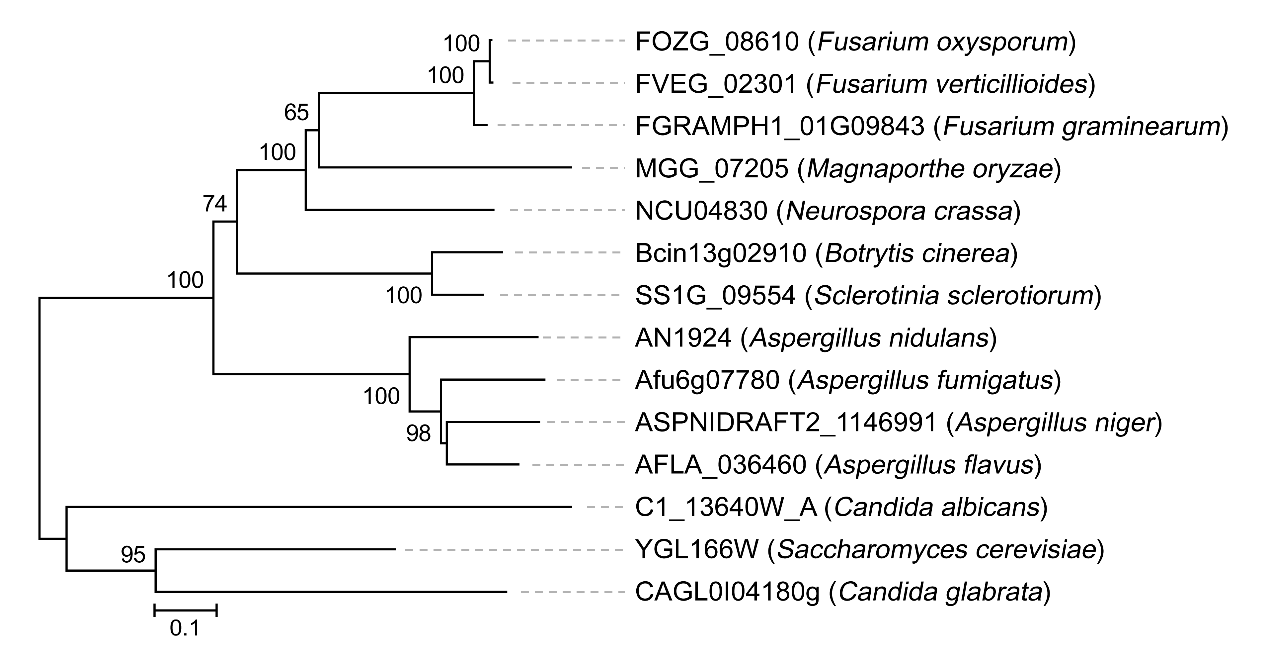
**

**B**

**
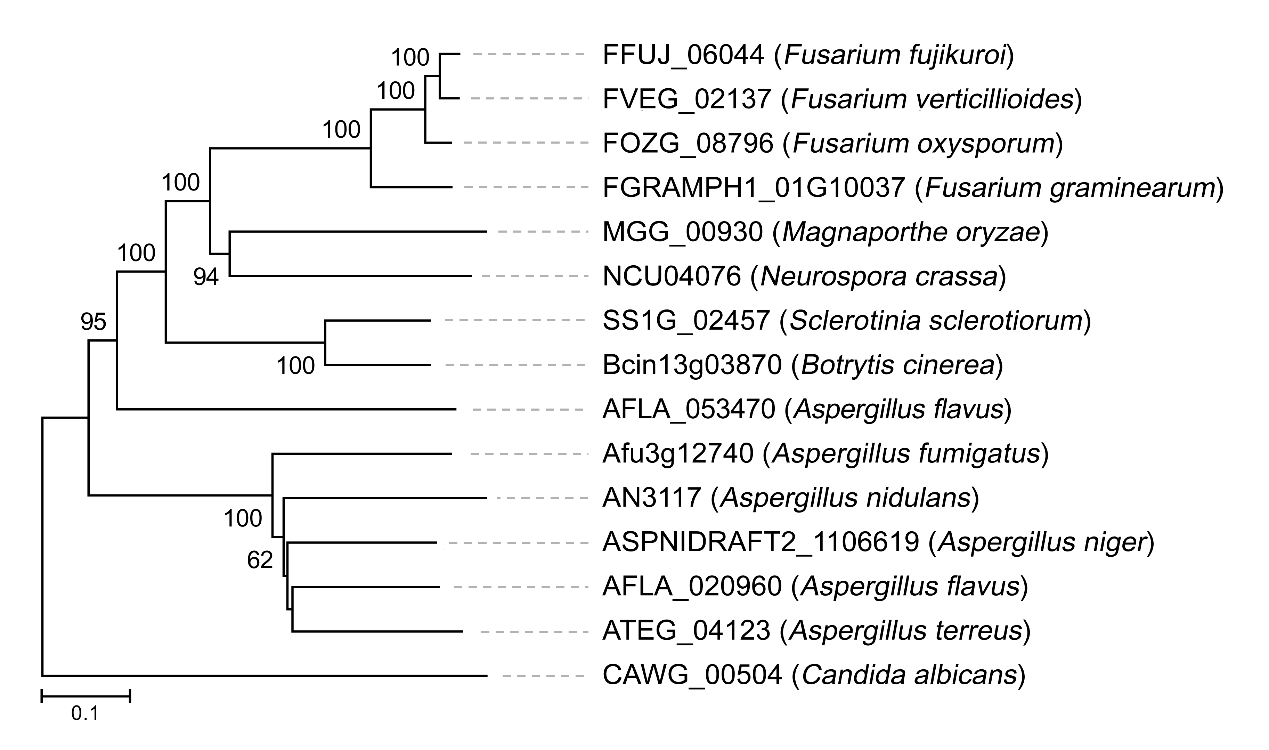
**

**C** **
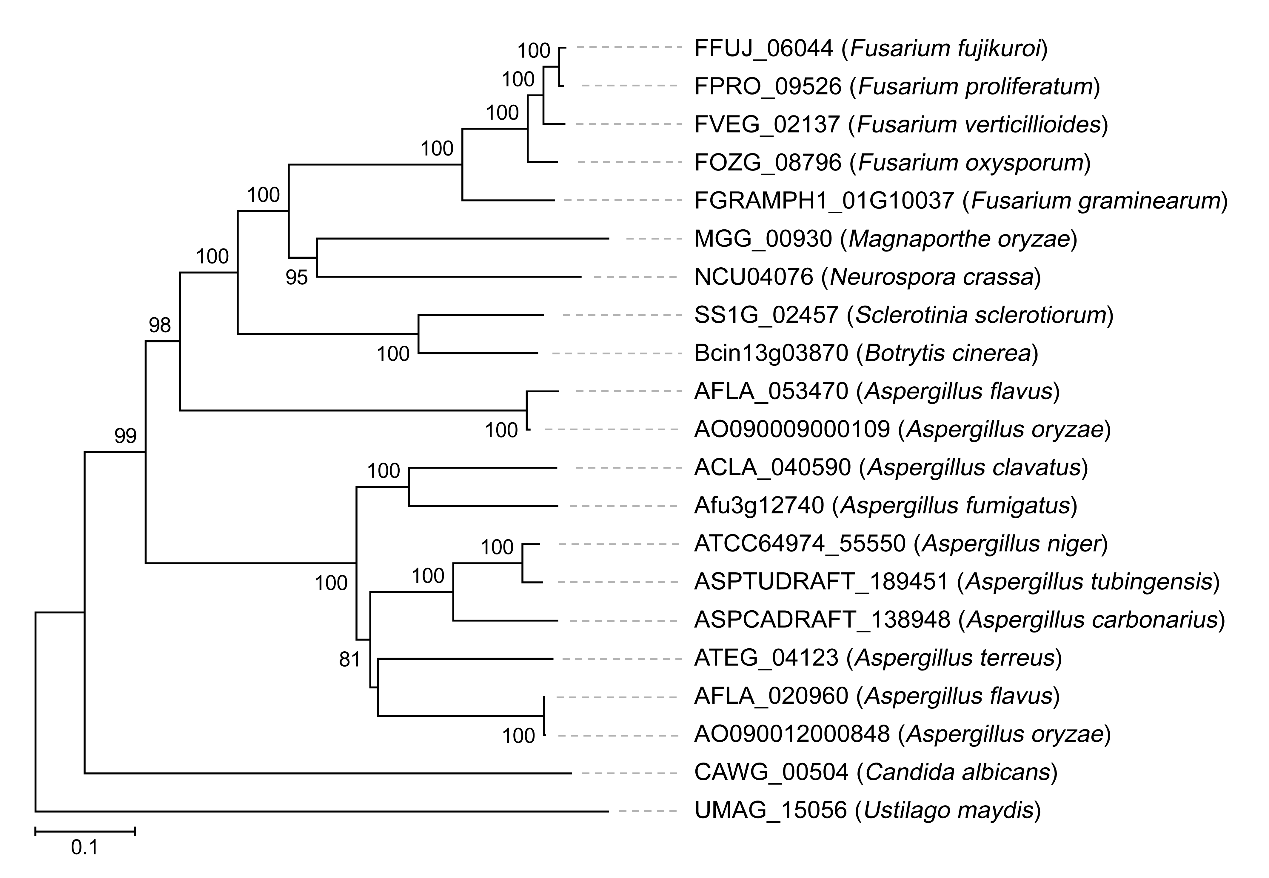
**

**FIGURE S2 |** Diagram for generation and verification for △FgAceA by Southern blotting analysis. **(A)** Diagram showing *Ban* II sites in the wild-type PH-1 *FgAceA* locus, and the locus after *in situ* replacement of hygromycin-resistance gene (*HPH*) or G418 sulfate-resistance gene (*NEO*), respectively. **(B)** Southern blotting analysis confirming the correct deletion for *FgAceA* gene by homologous integration as the expected band sizes at about 2.8 kb in wild-type PH-1, 4.7 kb in △FgAceA and 4.4 kb in △FgCrpA△FgAceA, and two bands (a 4.7 kb band and a size unknown band) in FgAceA^c^.


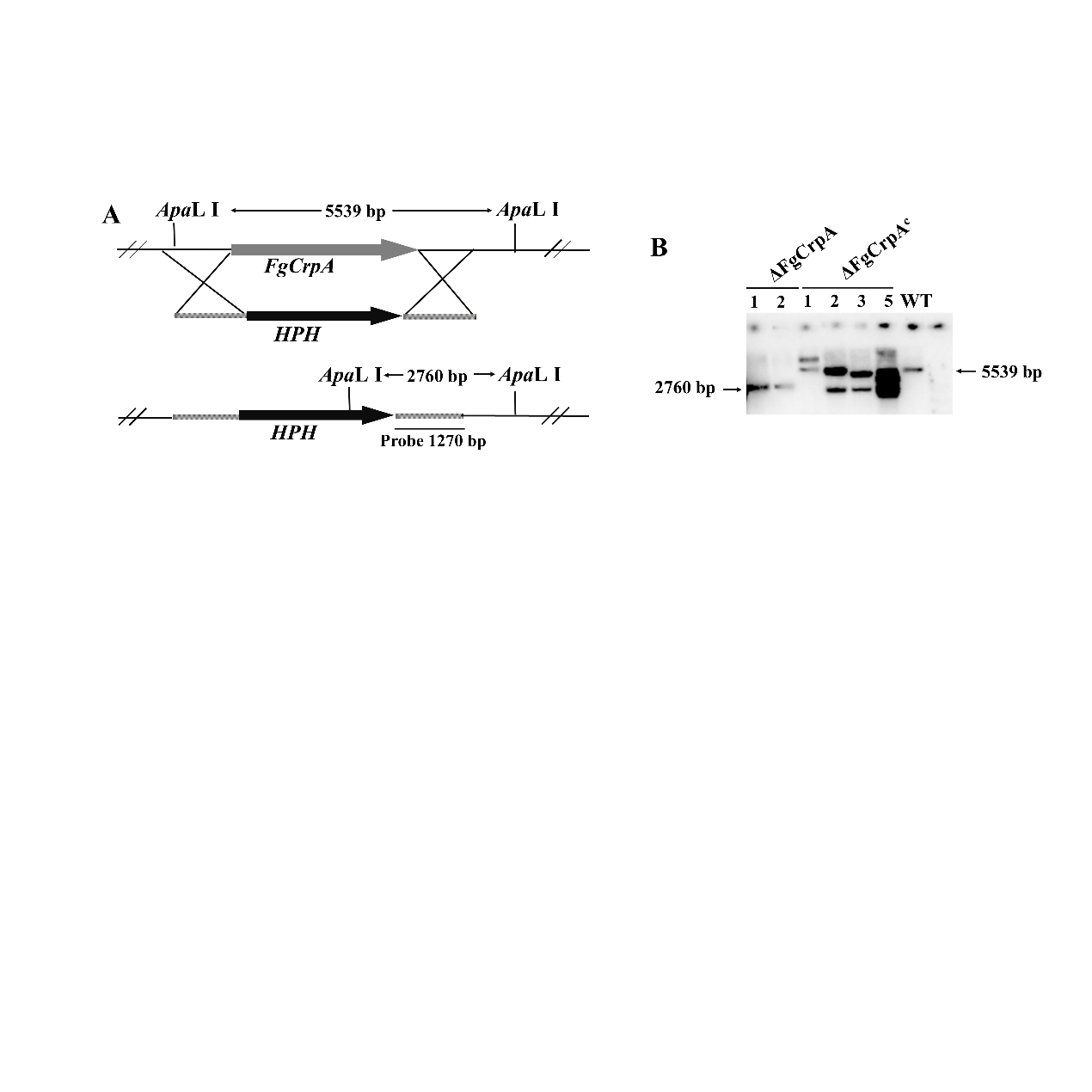


**FIGURE S3 |** Diagram for generation and verification for △FgCrpA by Southern blotting analysis. **(A)** Diagram showing *Apa*L I sites in the wild-type PH-1 *FgCrpA* locus, and the locus after *in situ* replacement of *HPH*, respectively. **(B)** Southern blotting analysis confirming the correct deletion for *FgCrpA* gene by homologous integration as the expected band sizes at about 2.8 kb in △FgCrpA, 5.5 kb in wild-type PH-1 and two bands (a 2.8 kb band and a size unknown band) in FgCrpA^c^.


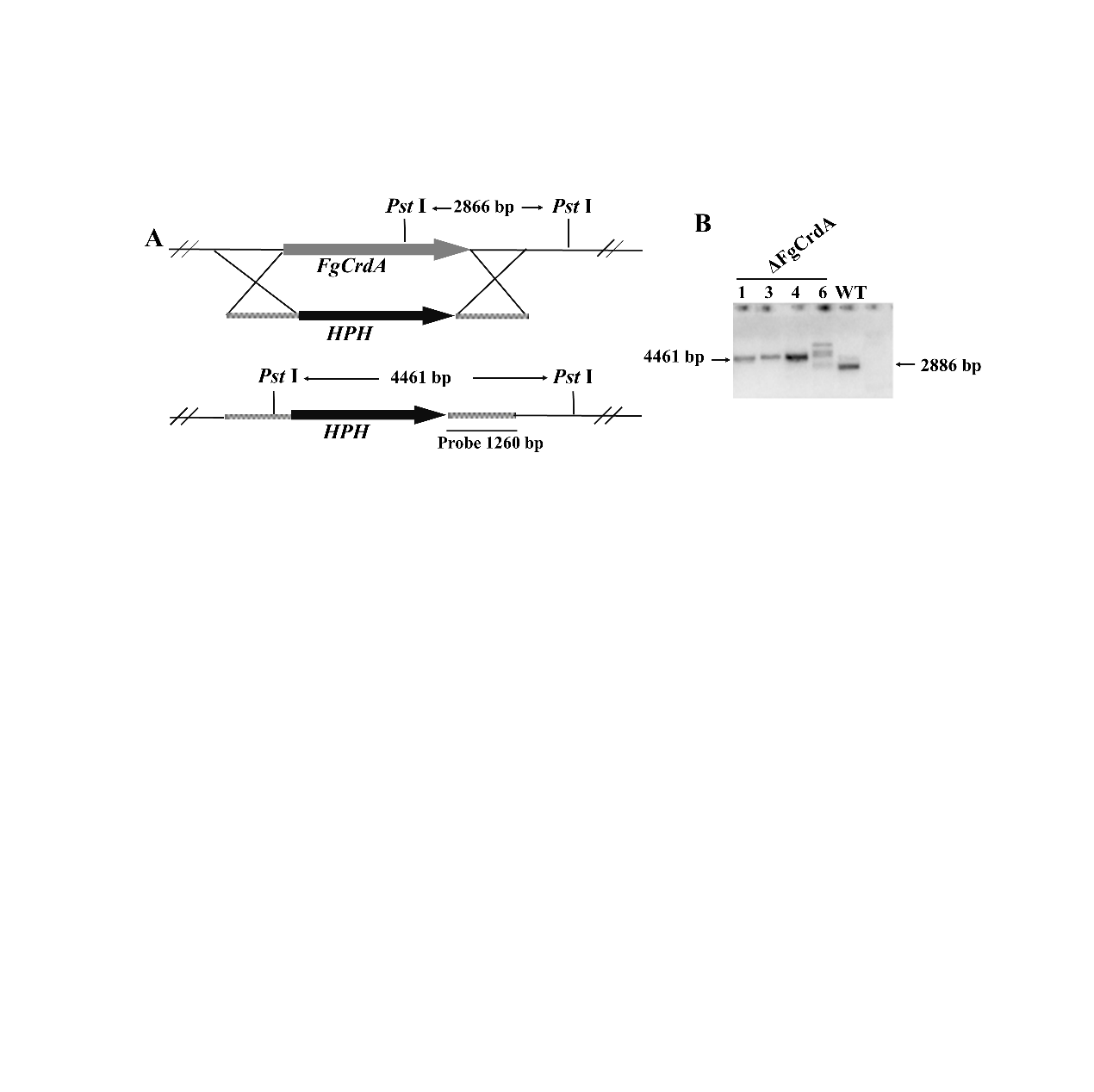


**FIGURE S4 |** Diagram for generation and verification for △FgCrdA by Southern blotting analysis. **(A)** Diagram showing *Pst* I sites in the wild-type PH-1 *FgCrdA* locus, and the locus after *in situ* replacement of *HPH*, respectively. **(B)** Southern blotting analysis confirming the correct deletion for *FgCrdA* gene by homologous integration as the expected band sizes at about 4.6 kb in △FgCrdA and 2.9 kb in wild-type PH-1.


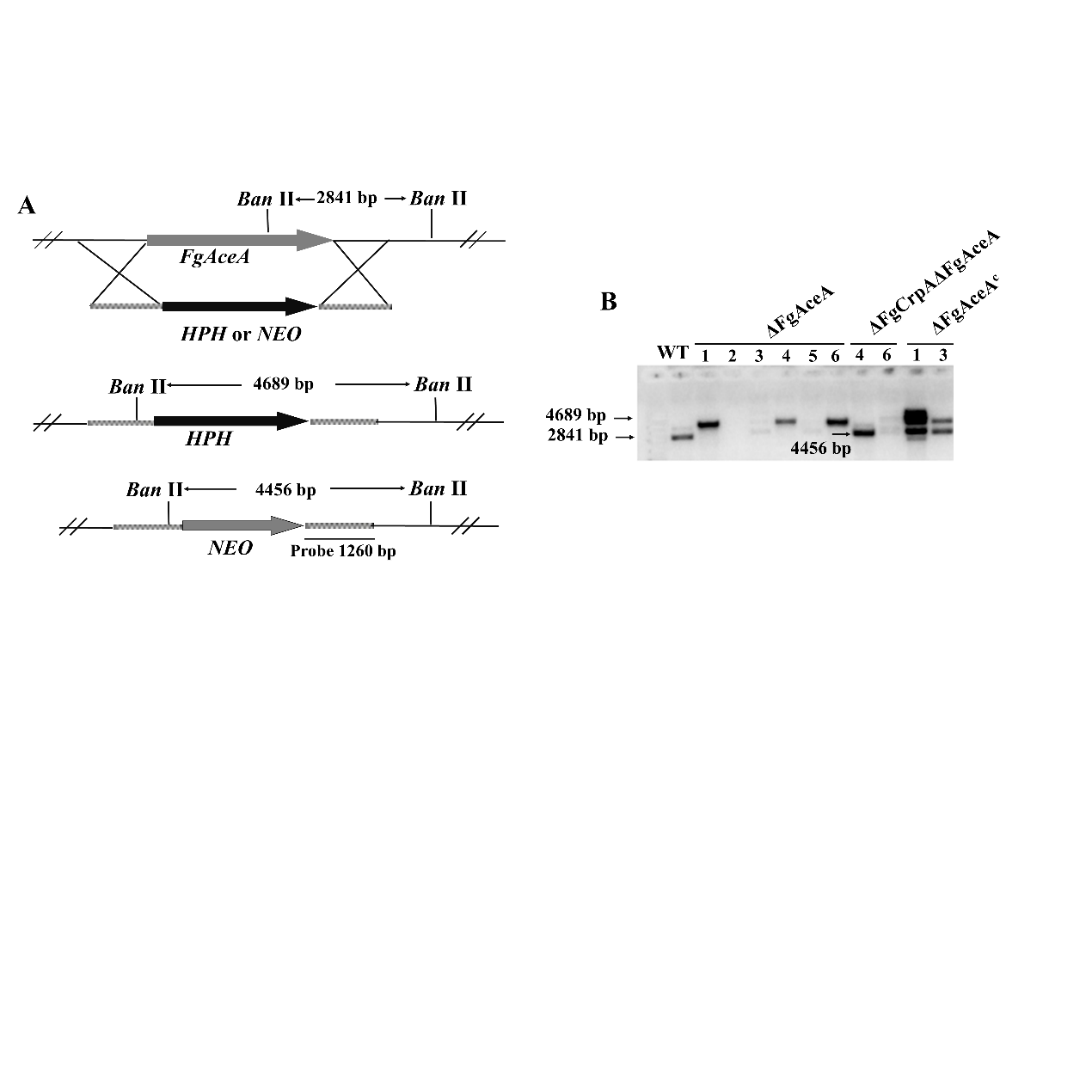


**FIGURE S5 |** Diagram for generation and verification for OE::FgCrpA by Southern blotting analysis. **(A)** Diagram showing *Sal* I sites in the wild-type PH-1 *FgCrpA* and its promoter locus, and the locus after insertion of G418 sulfate-resistance gene (*NEO*) and the constitutive prompter *gpdA* from *Aspergillus nidulans*, respectively. **(B)** Southern blotting analysis confirming the correct insertion for *FgCrpA* gene overexpression in wild-type strain PH-1 and △FgAceA as the expected band sizes at about 3.5 kb in OE::FgCrpA and 4.6 kb in wild-type PH-1 or △FgAceA.


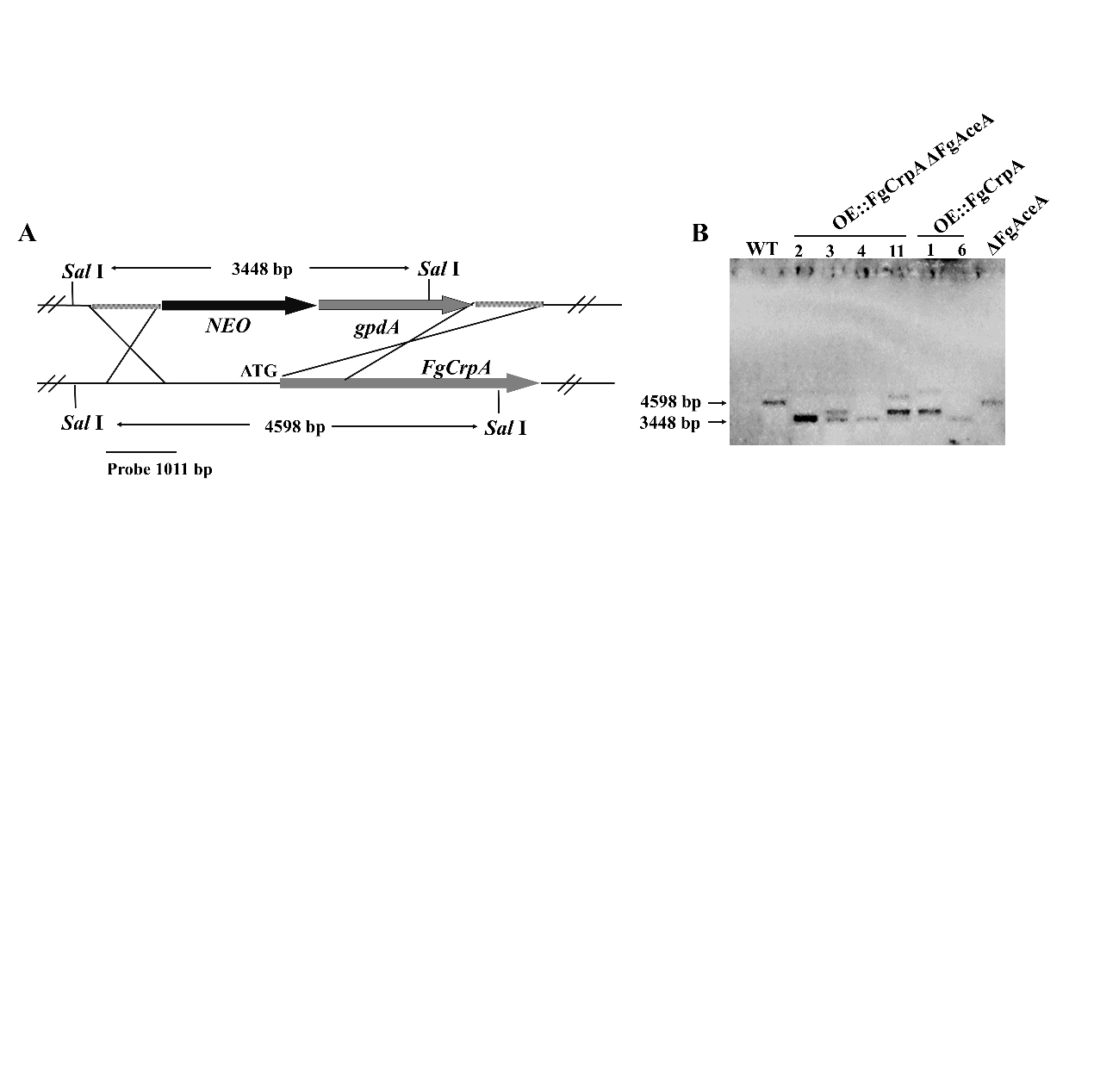


**Table S1.** Oligonucleotide primers used in this study and their relevant characteristics.

| **Primer** | **Sequence(5’-3’)** | | **Relevant characteristics** |
| --- | --- | --- | --- |
| A1 | GCGAGAAAGCAAGAATTCGA | | PCR primers for amplification of the upstream fragment of *FgAceA*. |
| A2 | CAAAATAGGCATTGATGTGTTGACCTCCGGGTGCGAACTGGTCTAAGAT | |  |
|  |  | |  |
| A3 | CTCGTCCGAGGGCAAAGGAATAGAGTAGGGTTTCGAATATCCTGGCGTA | | PCR primers for amplification of the downstream fragment *of FgAceA*. |
| A4 | ATGACTCCAAGGATCCGGG | |  |
|  |  | |  |
| A5 | CCAAGCGTATTCGACTGGGA | | PCR primers for identification of *FgAceA* disruption mutants. |
| A6 | TATGTATGTTGCCGCTGTTC | |  |
|  |  | |  |
| A7  A8 | TGCTGCTGATACTGCTGCTGA  CCCGGATAATTGTCATTGCT | | PCR primers for amplification of the upstream-*HPH*-downstream fragment for deletion of *FgAceA*. |
| B1 | TCGGAGGCCAACTTTTCTACT | | PCR primers for amplification of the upstream fragment of *FgCrpA*. |
| B2 | CAAAATAGGCATTGATGTGTTGACCTCCGACGCCGCACACATCAAA | |  |
|  |  | |  |
| B3 | CTCGTCCGAGGGCAAAGGAATAGAGTAGATGTCAAGCTGGACCCCGTAT | | PCR primers for amplification of the downstream fragment of *FgCrpA*. |
| B4 | CAAGGTAAGAAGCCAGGATCA | |  |
|  |  | |  |
| B5  B6 | GCAATCGAGCACAAAGATAG  GACTGCTCAATACCACACTG | | PCR primers for identification of *FgCrpA* disruption mutants. |
| B7  B8 | CACATCTTTACCTTGGTCGCA  TTCACTGCCTGACAATTGCA | | PCR primers for amplification of the upstream-*HPH*-downstream fragment for deletion of *FgCrpA*. |
| C1 | GTCAACACTCCTACTACCTG | | PCR primers for amplification of the upstream fragment of *FgCrdA*. |
| C2 | CCAAAATAGCATTGATGTGTTGACCTCCGTTCTACATACCAGCAGGAC | |  |
|  |  | |  |
| C3 | CTATCGCCTTCTTGACGAGTTCTTCTGA TTCAACCCTGCCAACGAGAT | | PCR primers for amplification of the downstream fragment of *FgCrdA*. |
| C4 | ATTTGGCTGATTTGTCCGGC | |  |
|  |  | |  |
| C5 | CGTAGCTCTGGAGCTGTAGATA | | PCR primers for the identification of *FgCrdA* disruption mutants. |
| C6 | CTTCTGCGCCATAACAAATGAA | |  |
|  |  | |  |
| C7  C8 | AACACTCCTACTACCTGCTG  CGGTTCGTGACAATGGATCG | | PCR primers for amplification of the upstream-*HPH*-downstream fragment for deletion of *FgCrdA*. |
| B9  B10 | ATAGTTCCTTTGCCTCTGTCG  TCGCTTATCATCTGATGGGA | | PCR primers for analysis of *FgCrpA* expression. |
| C9  C10 | CCCTCGATGCTCATGCC  GGCAGCCTTCTCACAAGT | | PCR primers for analysis of *FgCrdA* expression. |
|  |  | |  |
| B11  B12 | CCAAAATAGCATTGATGTGTTGACCTCCGACGCCGCACACATCAAA  CTATCGCCTTCTTGACGAGTTCTTCTGAATGTCAAGCTGGACCCCGTAT | | PCR primers for amplification of the downstream fragment with *NEO* marker adaptor of *FgCrpA*. |
|  |  | |  |
| A9  A10 | ACTCACTATAGGGCGAATTGGGTACTCAAATTGGTT ACCACGCCTCGCTGCTGCTG  CACCACCCCGGTGAACAGCTCCTCGCCCTTGCTCACCATGCCCCAAAAGCTGGGCAT | | PCR primers for amplification of the full-length fragment *of FgAceA* with its native promoter. |
| A11 | TTTCGTAGGAACCCAATCTTCAAAATGATAATTGATGGAGAAAAGT | | PCR primers for amplification of the full-length fragment *of FgAceA* with strong promoter. |
| A10 | CACCACCCCGGTGAACAGCTCCTCGCCCTTGCTCACCATGCCCCAAAAGCTGGGCAT | |  |
| B13  B14 | ACTCACTATAGGGCGAATTGGGTACTCAAATTGGTTGTTATGTCCTTTAGGCCTCAA  CACCACCCCGGTGAACAGCTCCTCGCCCTTGCTCACCTCCTCGTCGACTTCAATAC | | PCR primers for amplification of the full-length fragment *of* *FgCrpA* with its native promoter. |
| B15 | TTTCGTAGGAACCCAATCTTCAAAATGTCGTCTCTATCAACTGTTGT | | PCR primers for amplification of the full-length fragment *of FgCrpA* with strong promoter. |
| B14 | CACCACCCCGGTGAACAGCTCCTCGCCCTTGCTCACCTCCTCGTCGACTTCAATAC | |  |
|  |  | |  |
| B16 | TATCGCAGAAGGGAGGAAAT | | PCR primers for amplification of the upstream fragment for overexpression of *FgCrpA*.  PCR primers for amplification of the downstream fragment for overexpression of *FgCrpA*. |
| B17  B18  B19 | AGCATTGATGTGTTGACCTCCGAAGAACAATGCAGGTTGCA  CCCCGCTTGAGCAGACATCACCATGTCGTCTCTATCAACTGTTG  ATGCTTTGAGAGACTCATCAG | |  |
| B20 | TTGAGAGACTGATCGTTCCGT | | PCR primers for amplification of the cassette for overexpression of *FgCrpA*. |
| B21 | AGCATTAGGGGCATATCGAA | |  |
|  |  | |  |
| PKS12-RT-F | TGGTGTAGATGCTGTTCGTGT | | PCR primers for analysis of *PKS12* expression. |
| PKS12-RT-R | TGAACTTTTCGAGGACGGAT | |  |
|  |  | |  |
| AurJ-RT-F | AAAAAGCAGCCAAGGAGCAT | | PCR primers for analysis of *AurJ* expression. |
| AurJ-RT-R | TTCTGATGACACGCTCCCGTA | |  |
|  |  | |  |
| Gip1-RT-F | TGCGGTATCAGGTCACAAA | | PCR primers for analysis of *Gip1* expression. |
| Gip1-RT-R | ATCAAAGTCTCCCACCGTGAA | |  |
|  |  | |  |
| Gip2-RT-F | CACCAGCCCTACACCATCTAA | | PCR primers for analysis of *Gip2* expression. |
| Gip2-RT-R | TTTCCAAAGCGAGAAACAGC | |  |
|  |  | |  |
| AurF-RT-F  AurF-RT-R | ATCTTCAGTCTTGACCATCCC  TACCCAAGATGTTCTGGCAA | | PCR primers for analysis of *AurF* expression. |
| Fgtub-F | GGTAACCAAATCGGTGCTGCTTTC | | PCR primers for amplification of the reference gene actin, in real-time PCR. |
| Fgtub-R | TGCTTGGAGATCCACATTTG | |  |
|  |  | |  |
| Neo-F | GGAGGTCAACACATCAATGCT | | PCR primers for amplification of the G418 sulfate resistant gene *NEO*. |
| Neo-R | TCAGAAGAACTCGTCAAGAAG | |  |
|  |  | |  |
| Hph-F  Hph-R | GGAGGTCAACACATCAATGCCTATT  CTACTCTATTCCTTTGCCCT | | PCR primers for amplification of the hygromycin B resistant gene *HPH*. |
|  |  |  | |

**Table S2.** Expression of genes encoding putative copper-binding proteins revealed by RNA-seq.
